# Actual Associations between HLA Haplotype and Graves’ Disease Development

**DOI:** 10.3390/jcm11092492

**Published:** 2022-04-29

**Authors:** Katarzyna Zawadzka-Starczewska, Bogusław Tymoniuk, Bartłomiej Stasiak, Andrzej Lewiński, Magdalena Stasiak

**Affiliations:** 1 Department of Endocrinology and Metabolic Diseases, Polish Mother’s Memorial Hospital—Research Institute, 281/289 Rzgowska St., 93-338 Lodz, Poland; kasiula891@op.pl (K.Z.-S.); andrzej.lewinski@umed.lodz.pl (A.L.); 2 Department of Immunology, Rheumatology and Allergy, Medical University of Lodz, 251 Pomorska St., 92-213 Lodz, Poland; boguslaw.tymoniuk@umed.lodz.pl; 3 Institute of Information Technology, Lodz University of Technology, 215 Wolczanska St., 90-924 Lodz, Poland; bartlomiej.stasiak@p.lodz.pl; 4 Department of Endocrinology and Metabolic Diseases, Medical University of Lodz, 281/289 Rzgowska St., 93-338 Lodz, Poland

**Keywords:** Graves’ disease, human leukocyte antigen, HLA, susceptibility alleles

## Abstract

The association between HLA and the risk of Graves’ disease (GD) has been analyzed for many years. However, the results were often inconsistent and mostly regarded Asian populations. The purpose of our study was to perform HLA genotyping using a next-generation sequencing (NGS) method in Caucasians, to find out which alleles are eventually correlated with GD morbidity as well as which of them can be considered protective. *HLA-A, -B, -C, -DQB1, -DRB1* were genotyped using a next-generation sequencing method in 2376 persons, including 159 GD patients and 2217 healthy controls. We have demonstrated a significant association between the risk of GD and the following alleles: HLA-B*08:01, -B*39:06, -B*37:01, -C*07:01, -C*14:02, -C*03:02, -C*17:01, -DRB1*03:01, -DRB1*11:01, -DRB1*13:03, -DRB1*01:03, -DRB1*14:01, -DQB1*03:01, DQB1*02:01. The alleles HLA-B*39:06, -B*37:01, -C*14:02, -C*03:02, -C*17:01, -DRB1*14:01 are novel GD-associated, previously not-reported independent ones with no linkage disequilibrium with other high-risk alleles. On the other hand, the frequencies of HLA-B*07:02, -C*07:02, -C*03:04, DRB1*07:01, -DQB1*02:02, -DQB1*03:03 were significantly lower in GD compared to controls. This study demonstrated the actual relationships between HLA and GD based on the NGS method and provided a novel set of alleles as a reliable tool for an individual personalized risk assessment.

## 1. Introduction

Graves’ disease (GD) is an autoimmune thyroid disorder characterized by the production of specific antibodies against the thyrotropin (TSH) receptor. These TSH-receptor antibodies (TRAb) most frequently stimulate thyroid hormone production resulting in hyperthyroidism. However, TRAb may also block the TSH-receptor or have an ambivalent character [1]. The prevalence of GD in the Caucasian population is about 0.5–2.0% [1,2]. Similar to other autoimmune diseases, GD is usually triggered by environmental factors in genetically predisposed individuals [2,3]. Among genes associated with the immune response, human leukocyte antigen (HLA) genes have been found to be associated with autoimmune thyroid diseases (AITD), including GD [4]. Other genes such as cytotoxic T lymphocyte-associated factor 4 (*CTLA-4*), thyroglobulin (*Tg*) or *CD40* genes can also be associated with an increased risk of GD [5,6]. However, taking into account the relevance of the major histocompatibility complex (MHC) for immune responses and high polymorphism of HLA region, it seems to play a prominent role as a molecular background of GD [4].

Previous studies on HLA-related susceptibility to GD indicated the existence of significant ethnic differences [2,3,7,8,9,10,11,12]. Furthermore, the obtained results in either Asian or Caucasian populations are not consistent [3,7,8,9,10,11,12]. Most of the previously published studies applied much older methods, especially serological ones, which had significantly lower accuracy than the high-resolution next-generation sequencing (NGS) method used in our study. Moreover, the symbols of particular alleles assessed by the previously applied methods differ from those currently used, and some antigens previously denoted by one symbol are separated as several individual alleles when assessed by the high-resolution method. This fact undoubtedly has an important impact on the accuracy and consistency of the already published results.

Several studies conducted in the Asian population postulated the importance of the presence of HLA-B*46 in the development of GD [7,8,9], while others indicated a possible relationship with HLA-DRw8, -DQw4, -B5, Dw12 and -A11 antigens [13]. On the other hand, a Chinese study showed that the relationship between GD and HLA-B*46 concerned only men [14]. The same study found that the risk of GD was higher in patients with HLA-A*2, -Cw1, -DRB1*16:02, DRB1*03:01, DRB1*14:05, -DRB5*02, -DQB1*05:02, while the presence of -DRB1*15:01 and -DQB1*03:01 played a protective role [14]. Another study of Chinese patients observed the relationship between HLA-DR9 and -DQB1*03:03 in males [15]. Protective effects of HLA-DR12, previously described as an increased risk antigen, and of HLA-DQA1*04:01 were also postulated [15]. In turn, Japanese authors showed that the most important factor in the development of GD was the presence of HLA-DPB1*05:01 and/or HLA-A*2, with the risk being the highest in carriers of both of them [16]. In a Taiwanese study, HLA-A*02:07 was found to be a GD risk factor [17], while other Taiwanese authors showed that there was a correlation between GD and HLA-B*46:01, -DPB1*05:01, -DQB1*03:02, -DRB1*15:01 and -DRB1*16:02 [18]. Despite the apparent discrepancies, the results of a number of studies conducted on the Asian population are consistent in terms of the relationship between GD and the presence of alleles such as HLA-A*02:07, -B*46, -DRB1*08 or -DPB1*05:01 [6,17,18]. 

In Caucasians, studies are more scarce. Their results are consistent only with regard to the increased risk of GD in people with HLA-DRB1*03 and alleles remaining in linkage disequilibrium with HLA-DRB1*03 for this population, i.e., -DQA1*05: 01, -DQB1*02:01 [3,11,19,20]. However, it is well known that HLA-DRB1*03 is associated with an increased risk of all thyroid autoimmune diseases, not exclusively with GD. In regard to other alleles, Heward et al. postulated a possible role of HLA-DQB1*03:01/04 and -DQB1*02 in GD occurrence [3]. More recently, Vita et al. demonstrated significantly higher frequency of HLA-C*07, -C*17 and -DRB1*04 in patients with GD as compared to controls [2]. 

The small amount of data for the Caucasian population and the inconsistency between the results of individual studies, related mainly to different methods used and the size of the groups, left the HLA-related genetic basis of GD for the Caucasian population not satisfactorily explained. Therefore, there was a need to re-analyze and to compare HLA profiles in large groups of patients with GD and healthy controls using a modern high-resolution NGS method. By application of this method, our research group has recently demonstrated novel strong correlations between HLA and subacute thyroiditis (SAT), including not only the risk of SAT but also its clinical course [21,22,23,24,25]. It can be supposed that the relationship between GD and HLA is also much more complicated and includes more alleles than it was previously reported. 

The purpose of the study was to re-evaluate class I and class II MHC alleles in 159 patients with GD and 2217 healthy controls, and to clarify which HLA alleles are eventually associated with GD in the Caucasian population. Identification of an actual set of GD-related and GD-protective alleles would provide a novel reliable tool for the individual risk assessment and would constitute a great step in a development of personalized medicine. 

## 2. Materials and Methods

### 2.1. GD Group and Control Group

A total number of 2376 persons were included into the study, with 2217 healthy Polish hematopoietic stem cell donors with no medical history of thyroid disease, and 159 patients who were diagnosed with GD in the Department of Endocrinology and Metabolic Diseases, Polish Mother’s Memorial Hospital—Research Institute, Lodz, Poland, as well as in the Department-associated outpatient clinic. The size of the control group should be large enough to avoid any bias related to potential diseases which may appear in some members of this group in future, as well as to avoid any bias associated with random increased or decreased frequency of some alleles in a smaller control group.

### 2.2. HLA Typing Procedures

*HLA-A, -B, -C, -DQB1* and *-DRB1* were genotyped using a high-resolution NGS method. DNA was isolated from whole blood collected to the anticoagulant (EDTA)-containing tubes. Further sample preparation consisted of several steps, including long-range PCR, genomic library preparation, and sequencing. Amplicons were quantified by fluorescence detection method, balanced, pooled and enzymatically fragmented. Afterwards, end repair and A-tailing of DNA fragments was performed followed by index adapter ligation. Genomic library was cleaned up and denatured prior to loading on NGS Illumina Platform (NextSeq). We analyzed sequencing data with NGS HLA Genotyping software MIA FORA. The data were considered sufficient whenever the coverage reached 40.

### 2.3. Statistical Analysis

Allele frequencies were reported as absolute values and in percentages. The statistical significance of the differences between groups was evaluated by the chi-square test and by binomial logistic regression analysis, with *p* values ≤ 0.05 considered significant. The statistical analysis was carried out using Statistica v 13.1 software (Statsoft Polska, Kraków, Poland). 

### 2.4. Inclusion Criteria

In all patients included into the study, GD was diagnosed on the basis of standard criteria [1], including hyperthyroidism, elevated TRAb level, as well as typical ultrasound (US) pattern.

### 2.5. Biochemical and US Procedures

Serum levels of TSH, free triiodothyronine (FT3), free thyroxine (FT4) and TRAb were measured by the electrochemiluminescence immunoassay (ECLIA) with Cobas e601 analyzer (Roche Diagnostics, Indianapolis, IN, USA). Ultrasound examinations (US) were performed in every patient, using a 7–14 MHz linear transducer (Toshiba Aplio XG; Toshiba, Japan). 

### 2.6. Ethics Procedures

Informed consent for all performed procedures was obtained from all of the patients after a full explanation of the purpose and nature of all procedures used. The study was approved by the Ethics Committee of the Polish Mother’s Memorial Hospital—Research Institute, Lodz, Poland (approval code—108/2018). 

## 3. Results

The mean age of patients at diagnosis of GD was 43.88 ± 17.44 years, with a male to female ratio of 1:4.48. The diagnosis of GD was based on the laboratory results (Table 1). 

Statistically significant differences in the frequency of HLA alleles between patients with GD and control group were found with several alleles having higher frequency and others having lower frequency in GD as compared to controls. 

### 3.1. Alleles with Higher Frequencies in GD

**Figure 1:**
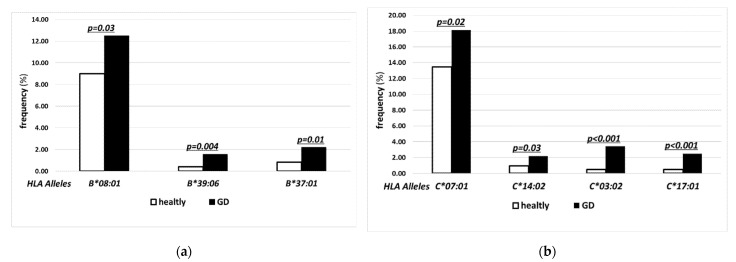
Frequencies (%) of human leukocyte antigen (HLA) over-represented alleles with statistically significant difference between control (open bars) and Graves’ disease (GD) patients (solid bars) for major histocompatibility complex (MHC) class I alleles: *HLA-B* (**a**) and *HLA-C* (**b**).

The alleles of higher frequency in GD as compared to controls were found in both MHC class I and class II. The differences were statistically significant for the following alleles of MHC class I: HLA-B*08:01 (12.5% vs. 9.0%), -B*39:06 (1.56% vs. 0.41%), -B*37:01 (2.19% vs. 0.83%) (Figure 1a), -C*07:01 (18.13% vs. 13.49%), -C*14:02 (2.19% vs. 0.95%), -C*03:02 (3.44% vs. 0.50%), -C*17:01 (2.50% vs. 0.50%) (Figure 1b). 

**Figure 2:**
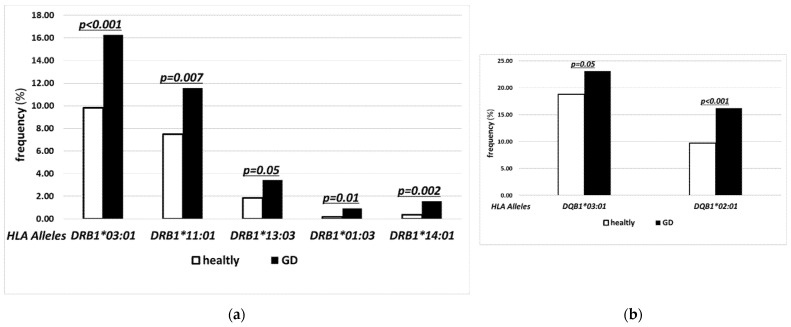
Frequencies (%) of human leukocyte antigen (HLA_ over-represented alleles with statistically significant difference between control (open bars) and Graves’ disease (GD) patients (solid bars) for major histocompatibility complex (MHC) class II alleles: *HLA-DRB1* (**a**) and *HLA-DQB1* (**b**).

For the MHC class II, significant differences in the frequencies were found for the following alleles: -DRB1*03:01 (16.25% vs. 9.83%), -DRB1*11:01 (11.56% vs. 7.49%), -DRB1*13:03 (3.44% vs. 1.87%), -DRB1*01:03 (0.94% vs. 0.20%), -DRB1*14:01 (1.56% vs. 0.36%) (Figure 2a), -DQB1*03:01 (23.13% vs. 18.83%), DQB1*02:01 (16.25% vs. 9.72%) (Figure 2b). 

### 3.2. Alleles with Lower Frequencies in GD

**Figure 3:**
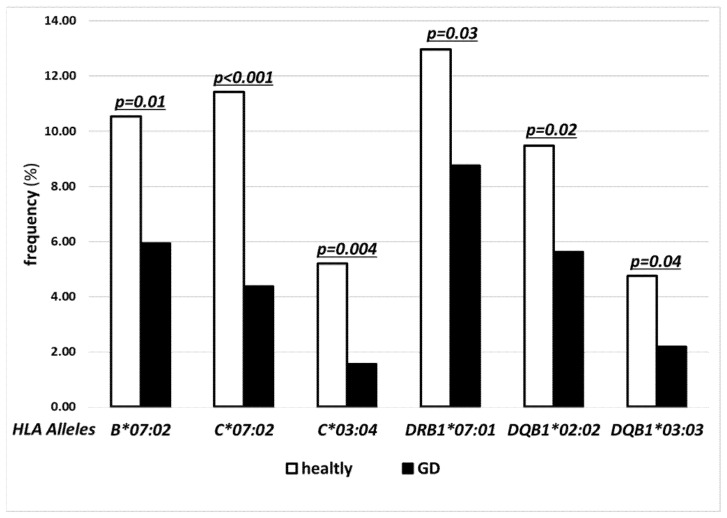
Frequencies (%) of human leukocyte antigen (HLA) under-represented alleles with statistical difference between controls (open bars) and Graves’ disease (GD) patients (solid bars) for both major histocompatibility complex (MHC) class I and class II alleles.

On the other hand, the frequencies of the following alleles were significantly lower in GD as compared to controls: HLA-B*07:02 (5.94% vs. 10.53%), -C*07:02 (4.38% vs. 11.41%), -C*03:04 (1.56% vs. 5.19%), -DRB1*07:01 (8.75% vs. 12.97%), -DQB1*02:02 (5.63% vs. 9.47%), -DQB1*03:03 (2.19% vs. 4.74%) (Figure 3). 

### 3.3. Significance of a Single High Risk Allele and of Co-Presence of Alleles

In 26 patients (16.4%), only one of the alleles described above as correlated to a high risk of GD was found. Among this group, the following alleles were found: HLA-B*39:06, -C*03:02, -C*07:01, -C*14:02, -DRB1*14:01 and -DQB1*03:01 in two (7.7%), three (11.5%), six (23.1%), one (3.8%), one (3.8%) and thirteen (50%) patients, respectively. 

In 33 patients (20.8%), two of the high-risk alleles were present. Among this group the co-presence of HLA-DRB1*11:01 and -DQB1*03:01 was observed the most frequently (45%). The co-presence of alleles which are not in linkage disequilibrium was observed in 9 patients (27.3%). Among this group, the most frequent was the combination of HLA-C*14:02 with -DQB1*03:01 (22.2%). 

Interestingly, allele HLA-B*08:01 was most frequently present with -DRB1*03:01 and -DQB1*02:01. A combination of the three alleles, -B*08:01, -DRB1*03:01- and DQB1*02:01, occurred in 36 of 159 GD patients (22%), while the co-presence of these three alleles was found only in 5.87% of the control group. In only three patients (1.9%), the HLA-B*08:01 allele occurred with other alleles (Table 2). In none of the patients did -B*08:01 occur as the only high-risk allele. 

## 4. Discussion

In the recent years, it has become more and more clear that GD is triggered by environmental factors such as infections, stress, smoking, etc., in genetically predisposed individuals [6,7]. This genetic susceptibility plays a critical role in the pathogenesis of GD and has been previously demonstrated to be HLA-dependent. Moreover, in both Asian and Caucasian populations, this genetic background was demonstrated to include MCH class I and class II. Therefore, the identification of alleles specifically related to GD seems to be a crucial step in the development of personalized medicine in regard to thyroid disorders. However, the results that have been reported for the last two decades in both populations are not consistent. Significant discrepancies in the results obtained by various authors can undoubtedly depend on the applied method. The use of high-resolution methods can change the results obtained with older methods. Genotyping methods of resolution that allow the achievement of allelic specificity is currently a gold standard of research that is expected to demonstrate high reliability, and to avoid method-dependent errors. Older, less accurate methods provide results for the entire allelic group, not for a particular allele. This may result in erroneous conclusions and discrepancies in the results of the studies depending on the method. According to the results obtained in a strictly controlled group of HLA typing performed for the purposes of bone marrow transplantation between 1996 and 2011, 29.1% discrepancies were found between older methods and NGS method [26] which was applied in the present study. Another important example of the significance of the genotyping method is HLA-B*27 test commonly used to genetically confirm a diagnosis of ankylosing spondylitis. It has been recently demonstrated that HLA typing methods used so far gave insufficiently precise results, and two alleles, HLA-B*27:06 and HLA-B*27:09, are probably not associated with the disease [27]. These examples clearly present the importance of the method and a possible role of a method-dependent factor in the inconsistency of the previous results. 

To date, to the best of our knowledge, the present study included the largest Caucasian cohort to whom a modern high-resolution method was applied to analyze both MCH classes. Thus, this study can summarize and clarify the actual HLA-related genetic background of GD. 

The present study has confirmed a strong correlation between GD and HLA-B*08:01, DRB1*03:01, -DQB1*02:01 (Figure 1a and Figure 2a,b). Our observation is consistent with the previous reports [2,3,11,12,19,20]. Interestingly, in our study, HLA-B*08:01 was accompanied by these two alleles in most cases of its presence although it belongs to a different MCH class (Table 2). The co-presence of these three alleles in GD group was 4 times more frequent than in controls. Additionally, HLA-DRB1*03:01 and -DQB1*02:01 were rarely present without HLA-B*08:01 (Table 2). Such a close unexpected association between HLA-B*08:01 and MCH class II alleles was previously postulated in the Caucasian population [28], but the present study has confirmed it and demonstrated its strength for GD patients for the first time. This finding sheds a new light on the possible linkage disequilibrium between alleles from different MCH classes. Moreover, it indicates the existence of specific mechanism of impact augmentation between these alleles in GD. 

The currently discussed group of linkage disequilibrium includes also HLA-DQA1*05:01 [27,29,30], previously reported as GD high risk allele [2,3,19]. We did not perform comparison of frequencies of HLA-DQA1*05:01 between our groups because *HLA-DQA1* alleles are not reported for transplantation purposes and results performed using NGS method are unavailable either for our control group or for any other representatively large cohort in Poland. Comparison with any population with available lower resolution results could introduce unacceptable bias, as the main strength of the present study is the application of the most reliable method. However, a strong linkage disequilibrium between HLA-DQA1*05:01 and DRB1*03:01, DQB1*02:01, as well as other alleles demonstrated as high-risk ones in the present study, i.e., DRB1*01:03, DQB1*03:01 and DRB1*13:03 (Figure 2) seems to confirm previous findings of HLA-DQA1*05:01 being a GD risk allele [2,3,19]. The potential role of HLA-DQB1*03:01 was previously postulated by Heward et al. [3]. Similar to our study, Martin et al. observed significantly higher frequency of HLA-DRB1*11:01 and -DRB1*13:03 [31]. Our study has confirmed the role of these alleles as well as has further supported the role of HLA-DQA1*05:01 because of linkage disequilibrium between either HLA-DRB1*11:01 or -DRB1*13:03 and HLA-DQA1*05:01 [29]. Both of these *HLA-DRB1* alleles are also in linkage disequilibrium with HLA-DQB1*03:01 which has been confirmed in the present study as high-risk allele. To the best of our knowledge, this is the first report presenting strong susceptibility to GD related to HLA-DRB1*01:03—the allele that is also in linkage disequilibrium with HLA-DQA1*05:01 and -DQB1*03:01 [29]. 

Our study also demonstrated that the risk of GD is significantly increased in patients with a presence of HLA-C*07:01, being in linkage disequilibrium with previously discussed -B*08:01 [32,33]. It is worth mentioning that the importance of HLA-B*08:01 and -C*07:01 was previously postulated in the literature, mostly as -B*08 and -C*07 with application of lower resolution methods and two-digit results [2,34]. Our study has confirmed this association for HLA-B*08:01 and -C*07:01 (Figure 1a,b). When analyzing the above-described associations, one should keep in mind that susceptibility associated with alleles being in linkage disequilibrium cannot be considered as fully independent. However, the single presence of any of them can be correlated with the risk of GD. 

This study has confirmed the correlation of GD with HLA-C*17:01 postulated before by Vita et al. [2]. Our study based on NGS method has demonstrated even stronger statistical significance than previously reported (Figure 1b). No linkage disequilibrium has been reported between HLA-C*17:01 and other high-risk alleles [32,33], so HLA-C*17:01 should be considered an independent one. 

The most important finding of our present study is the significance of novel alleles which have been reported here as GD-related for the first time. This group includes HLA-B*37:01, -B*39:06, -C03:02, -C14:02, and -DRB1*14:01 (Figure 1a,b and Figure 2a). These alleles belong to both I and II MCH classes and are not in linkage disequilibrium either with each other or with any previously discussed GD-related alleles [27,32,33]. Thus, their significance for the pathogenesis of GD is particularly relevant. 

Similar to Vita et al. [2], we have not confirmed a higher frequency of HLA-DRB1*08 previously postulated in North American and British Caucasians [34,35]. The specificity of the applied method may have played the most important role in these differences. 

Our study has demonstrated strong association of GD with the presence of several alleles belonging to both MCH classes. Strong impact of any of them on the risk of GD can be additionally supported by our finding that the presence of a single allele from the high-risk group is sufficient to induce GD. In the present study, alleles HLA-B*39:06, -C*03:02, -C*07:01, -C*14:02, -DRB1*14:01 and -DQB1*03:01 were present as a single allele in GD patients. Most of them, i.e., HLA-B*39:06, -C*03:02, -C*14:02, -DRB1*14:01, have been reported for the first time as high-risk alleles in the present study. It is worth underlining that among patients with two high risk alleles, the co-presence of HLA-DRB1*11:01 and -DQB1*03:01, being in linkage disequilibrium was the most common. However, in 27.3% of the patients, the co-present alleles were totally independent, with HLA-C*14:02—newly reported in the present paper—being the most frequent independent allele. 

Predisposition for autoimmune disorders other than GD can also be HLA-related. Hashimoto’s thyroiditis (HT) and GD share a variety of common etiological and pathophysiological factors, including HLA-based predisposition, a trend to aggregate in the same families or even to coexist in the same gland [36]. Moreover, several reports suggested the existence of a continuum between HT and GD [37,38,39]. In our GD group, only four patients had preceding Hashimoto’s thyroiditis, and in all of them HLA-DQB1*02:01 was present. This allele was demonstrated in our results as one of the alleles related to high risk of GD and it is in linkage disequilibrium with -*DRB1:03:01*—an allele typical for thyroid autoimmunity. Interestingly, the co-presence of these two alleles was found in all patients with coexistence of GD and non-thyroidal autoimmunity (i.e., in two patients with Addison’s disease and two patients with diabetes type 1). These subgroups were too small to obtain any statistical results, but this phenomenon requires further analysis.

It was previously postulated that the presence of some HLA alleles may play a protective role against GD. Similar to the results on high-risk alleles, most of the papers published so far regard the Asian population. Yin et al. postulated that HLA-B*33 can protect against GD [40], while Mehraji et al. demonstrated that HLA-DQB1*02:01 and -DQA1*02:01 play the protective role [41]. The results of studies in the Korean population did not confirm the previous findings and revealed that alleles HLA-DRB1*01:01, DRB1-*07:01, -DRB1*12:02 and -DRB1*13:02 were the protective ones [42] On the other hand, other Korean group confirmed the protective role of HLA-DQA1*02:01 and -DQB1*02:01 as well as HLA-DRB1*12, and additionally postulated the significance of -DQA1*06:01 and -DQB1*03:01 [43]. In the Chinese population, Cavan et al. postulated the significance of -DQA1*04:01 and confirmed previously reported protective role of HLA-DRB1*12 and -DQB1*03:01 [15]. The results obtained by the different research groups were inconsistent and it is difficult to unambiguously distinguish the actually protective alleles. The main candidates in the Asian population seem to be HLA-DRB1*12:02, -DQB1*02:01 and DQB1*03:01.

A similar situation of inconsistent results regards the Caucasian population, and in addition, the results are more scarce. Therefore, our present study aimed also to compare GD and control cohorts with regard to potentially protective alleles. We confirmed the previously reported significantly less frequent presence of HLA-DRB1*07:01, [12,34,35] and -DQB1*02:02 [34] in patients with GD as compared to healthy controls (Figure 3). However, in the last study, the significance of -DQB1*02 was postulated in two-digit presentation [34]. We have refined and clarified this finding using the NGS method. Our study revealed also lower frequency of HLA-DQB1*03:03 in GD individuals (Figure 3). It has to be underlined that HLA-DQB1*02:02 and -DQB1*03:03 are in linkage disequilibrium with -DRB1*07:01, together with the previously postulated -DQA1*02:01 [12,27].

The presence of these protective alleles can play a very important role in GD development. Proteins related to HLA-DRB1*03:01 and its linkage disequilibrium alleles differ from those related to -DRB1*07:01 and its linkage disequilibrium-related alleles at position β74, a crucial site in the binding pocket of the HLA allele, the residue being arginine and glutamine, respectively [2,34]. It has been hypothesized that in patients with the co-presence of these two alleles, HLA-DRB1*07:01 can cancel out the GD-susceptibility associated with -DRB1*03:01 [2,34].

Similar to our findings on novel high-risk alleles, we have also demonstrated novel, previously not reported, protective associations. In the present study, the frequencies of HLA-B*07:02 and -C*07:02 were significantly lower in the GD cohort than in the control group (Figure 3). These alleles cannot be considered independent as there is also a linkage disequilibrium between them. However, as it was previously underlined, the presence of any of them can potentially be sufficient as a protective factor. Moreover, another novel independent allele was revealed as protective, i.e., -HLA-C*03:04, with a high statistical significance of *p* = 0.004 (Figure 3). This allele is not in linkage disequilibrium with any other potentially protective allele.

The differences in the results between Caucasian and Asian populations with regard to the MCH class II alleles, which are in linkage disequilibrium with HLA-DQA1*05:01 can be considered unexpected. Among all of these alleles, only -DRB1*03:01 was proved to be GD-related in the Asian population [14]. However, all of the above-described linkage disequilibrium-based correlations are common not exclusively for the Caucasian population but for all analyzed populations, including Asians [29]. Therefore, the question arises why the correlations present in Caucasians are absent in Asians, in whom completely different alleles are considered as high risk of GD. Furthermore, taking into account our present results and the literature data, we can observe a phenomenon of entirely opposite roles of HLA-DQB1*02:01 and -DQB1*03:01 in these populations. These alleles have been demonstrated as high risk in Caucasians in the present study and in some previous ones [3]. However, they are reported as protective in Asian studies [15,42,43]. Therefore, although HLA-DRB1*03:01 has been demonstrated to be a high-risk allele in both populations, -DQB1*03:01 being in linkage disequilibrium with -DRB1*03:01 in both populations, is a high-risk allele in Caucasians and a protective allele in Asians. This fact demonstrates the impact of other factors influencing GD risk in the Asian population and points out the necessity for further analysis of this phenomenon. 

## 5. Conclusions

The present study has demonstrated the actual associations between HLA haplotype and GD. The application of the NGS method to genotype both MCH I and II classes in large groups of patients and controls has clarified many discrepancies present in the previous results possibly due to a lack of allelic specificity and/or the size of the groups being too small. A significant association was found between the risk of GD and the following alleles: HLA-B*08:01, -B*39:06, -B*37:01, -C*07:01, -C*14:02, -C*03:02, -C*17:01, -DRB1*03:01, -DRB1*11:01, -DRB1*13:03, -DRB1*01:03, -DRB1*14:01, -DQB1*03:01, DQB1*02:01. Among these alleles, -B*39:06, -B*37:01, -C*14:02, -C*03:02, -C*17:01, -DRB1*14:01 are novel, previously not-reported, independent alleles with no known linkage disequilibrium with other high-risk alleles. On the other hand, the frequencies of HLA-B*07:02, -C*07:02, -C*03:04, -DRB1*07:01, -DQB1*02:02, -DQB1*03:03 were significantly lower in GD compared to controls, with the first three alleles being reported as protective for the first time. These alleles can be considered protective. This study has provided a novel set of alleles as a reliable tool for individual risk assessment. The identification of alleles which in a particular population are associated with GD and which play a protective role is an essential step in the development of personalized medicine. 

## Figures and Tables

**Table 1:** Laboratory characteristics of the Graves’ disease (GD) group.

Parameter (Reference Range and Units)	Mean ± SD	Median
TSH (0.27–4.2 µIU/mL)	0.14 ± 0.43	0.05
FT4 (0.9–1.7 ng/dL)	3.35 ± 2.39	2.33
FT3 (2.0–4.4 pg/mL)	11.07 ± 8.38	7.86
TRAb (<1.7 IU/L)	15.04 ± 13.62	10.12

Abbreviations: FT3, free triiodothyronine; FT4, free thyroxine; SD, standard deviation; TRAb, TSH receptor antibodies; TSH, thyrotropin.

**Table 2:** Frequencies and linkage disequilibrium of three-locus *HLA-B-DRB1-DQB1* haplotypes in Graves’ disease (GD) patients depending on the presence of the HLA-B*08:01 allele.

HLA Haplotype	Haplotype Frequency
B*08:01- DRB1*03:01- DQB1*02:01	22% [*n* = 36]
B*XX:XX- DRB1*03:01- DQB1*02:01	6.6% [*n* = 11]
B*08:01- DRB1*XX:XX- DQB1*XX:XX	1.9% [*n* = 3]

B*XX:XX—allele other than -B*08:01; DRB1*XX:XX- DQB1*XX:XX—alleles other than -DRB1*03:01,- DQB1*02:01.

## Data Availability

The source data are available on reasonable request from the corresponding author.

## Abbreviations

AITD	autoimmune thyroid diseases
ATA	American Thyroid Association
CD40	cluster of differentiation 40
CTLA-4	cytotoxic T lymphocyte-associated factor 4
EDTA	ethylenediaminetetraacetic acid (anticoagulant)
FT3	free triiodothyronine
FT4	free thyroxine
GD	Graves’ disease
HLA	human leukocyte antigens
MHC	major histocompatibility complex
NGS	next-generation sequencing
SAT	subacute thyroiditis
Tg	thyroglobulin
TRAb	TSH-receptor antibodies
TSH	thyroid stimulating hormone (thyrotropin)
US	ultrasound
